# Colchicine for community-treated patients with COVID-19 (COLCORONA): a phase 3, randomised, double-blinded, adaptive, placebo-controlled, multicentre trial

**DOI:** 10.1016/S2213-2600(21)00222-8

**Published:** 2021-08

**Authors:** Jean-Claude Tardif, Nadia Bouabdallaoui, Philippe L L'Allier, Daniel Gaudet, Binita Shah, Michael H Pillinger, Jose Lopez-Sendon, Protasio da Luz, Lucie Verret, Sylvia Audet, Jocelyn Dupuis, André Denault, Martin Pelletier, Philippe A Tessier, Sarah Samson, Denis Fortin, Jean-Daniel Tardif, David Busseuil, Elisabeth Goulet, Chantal Lacoste, Anick Dubois, Avni Y Joshi, David D Waters, Priscilla Hsue, Norman E Lepor, Frédéric Lesage, Nicolas Sainturet, Eve Roy-Clavel, Zohar Bassevitch, Andreas Orfanos, Gabriela Stamatescu, Jean C Grégoire, Lambert Busque, Christian Lavallée, Pierre-Olivier Hétu, Jean-Sébastien Paquette, Spyridon G Deftereos, Sylvie Levesque, Mariève Cossette, Anna Nozza, Malorie Chabot-Blanchet, Marie-Pierre Dubé, Marie-Claude Guertin, Guy Boivin, for the COLCORONA Investigators

**Affiliations:** a Montreal Heart Institute, Université de Montréal, Montreal, QC, Canada; b Ecogene-21, Université de Montréal, Montreal, QC, Canada; c Department of Medicine, Université de Montréal, Montreal, QC, Canada; d New York University Grossman School of Medicine, New York, NY, USA; e H La Paz, IdiPaz, UAM, Ciber-CV, Madrid, Spain; f Instituto do Coração, Hospital das Clínicas, Faculdade de Medicina, Universidade de São Paulo, São Paulo, Brasil; g Centre Hospitalier Universitaire de Québec, Université Laval, Quebec City, QC, Canada; h Mayo Clinic, Rochester, MN, USA; i San Francisco General Hospital, San Francisco, CA, USA; j Cedars-Sinai Heart Institute, Geffen School of Medicine-UCLA, Los Angeles, CA, USA; k Montréal Health Innovations Coordinating Center, Montreal, QC, Canada; l Hôpital Maisonneuve-Rosemont, Université de Montréal, Montreal, QC, Canada; m Centre Hospitalier de l'Université de Montréal, Montreal, QC, Canada; n Université Laval, Quebec City, QC, Canada; o Second Department of Cardiology, National and Kapodistrian University of Athens, Athens, Greece

## Abstract

**Background:**

Evidence suggests a role for excessive inflammation in COVID-19 complications. Colchicine is an oral anti-inflammatory medication beneficial in gout, pericarditis, and coronary disease. We aimed to investigate the effect of colchicine on the composite of COVID-19-related death or hospital admission.

**Methods:**

The present study is a phase 3, randomised, double-blind, adaptive, placebo-controlled, multicentre trial. The study was done in Brazil, Canada, Greece, South Africa, Spain, and the USA, and was led by the Montreal Heart Institute. Patients with COVID-19 diagnosed by PCR testing or clinical criteria who were not being treated in hospital were eligible if they were at least 40 years old and had at least one high-risk characteristic. The randomisation list was computer-generated by an unmasked biostatistician, and masked randomisation was centralised and done electronically through an automated interactive web-response system. The allocation sequence was unstratified and used a 1:1 ratio with a blocking schema and block sizes of six. Patients were randomly assigned to receive orally administered colchicine (0·5 mg twice per day for 3 days and then once per day for 27 days thereafter) or matching placebo. The primary efficacy endpoint was the composite of death or hospital admission for COVID-19. Vital status at the end of the study was available for 97·9% of patients. The analyses were done according to the intention-to-treat principle. The COLCORONA trial is registered with ClinicalTrials.gov (NCT04322682) and is now closed to new participants.

**Findings:**

Trial enrolment began in March 23, 2020, and was completed in Dec 22, 2020. A total of 4488 patients (53·9% women; median age 54·0 years, IQR 47·0–61·0) were enrolled and 2235 patients were randomly assigned to colchicine and 2253 to placebo. The primary endpoint occurred in 104 (4·7%) of 2235 patients in the colchicine group and 131 (5·8%) of 2253 patients in the placebo group (odds ratio [OR] 0·79, 95·1% CI 0·61–1·03; p=0·081). Among the 4159 patients with PCR-confirmed COVID-19, the primary endpoint occurred in 96 (4·6%) of 2075 patients in the colchicine group and 126 (6·0%) of 2084 patients in the placebo group (OR 0·75, 0·57–0·99; p=0·042). Serious adverse events were reported in 108 (4·9%) of 2195 patients in the colchicine group and 139 (6·3%) of 2217 patients in the placebo group (p=0·051); pneumonia occurred in 63 (2·9%) of 2195 patients in the colchicine group and 92 (4·1%) of 2217 patients in the placebo group (p=0·021). Diarrhoea was reported in 300 (13·7%) of 2195 patients in the colchicine group and 161 (7·3%) of 2217 patients in the placebo group (p<0·0001).

**Interpretation:**

In community-treated patients including those without a mandatory diagnostic test, the effect of colchicine on COVID-19-related clinical events was not statistically significant. Among patients with PCR-confirmed COVID-19, colchicine led to a lower rate of the composite of death or hospital admission than placebo. Given the absence of orally administered therapies to prevent COVID-19 complications in community-treated patients and the benefit of colchicine in patients with PCR-proven COVID-19, this safe and inexpensive anti-inflammatory agent could be considered for use in those at risk of complications. Notwithstanding these considerations, replication in other studies of PCR-positive community-treated patients is recommended.

**Funding:**

The Government of Quebec, the Bill & Melinda Gates Foundation, the National Heart, Lung, and Blood Institute of the US National Institutes of Health, the Montreal Heart Institute Foundation, the NYU Grossman School of Medicine, the Rudin Family Foundation, and philanthropist Sophie Desmarais.

## Introduction

Accumulating evidence suggests that some patients with COVID-19 have excessive inflammation.^1^ Treatment of this exaggerated inflammatory response has been advocated to address the immediate need to reduce the risk of complications.^1^, ^2^ The steroid dexamethasone reduces mortality in patients admitted to hospital with COVID-19, but only if they receive mechanical ventilation or supplemental oxygen.^3^ In the open-label STOIC trial,^4^ the inhaled steroid budesonide decreased the requirement for urgent medical care in patients with early COVID-19. In addition, the anti-interleukin (IL)-6-receptor antibody tocilizumab was shown to reduce the likelihood of progression to mechanical ventilation in patients admitted and treated in hospital for COVID-19 pneumonia.^5^ The IL-1 receptor antagonist anakinra might also be effective in some patients with COVID-19.^6^

SARS-CoV, which is closely related to the SARS-CoV-2 virus responsible for COVID-19, has been shown to activate the NLR-family pyrin domain-containing 3 (NLRP3) inflammasome.^7^ Activation of the NLRP3 inflammasome by SARS-CoV-2 has been shown in lung tissues of patients with COVID-19.^8^ This intracellular complex activates several ILs, which then trigger an inflammatory cascade. Given that elevated concentrations of IL-1β and IL-6 are associated with adverse clinical outcomes in COVID-19,^9^, ^10^ targeting the NLRP3 inflammasome (which is responsible for the maturation and secretion of IL-1β) might reduce related complications in patients at risk of cytokine activation.

**Research in context**

**Evidence before this study**

We searched MEDLINE (PubMed), Embase, and Cochrane central to identify studies investigating the role of the host inflammatory response in COVID-19 complications and randomised controlled trials (RCTs) of anti-inflammatory agents aimed at preventing this response, from inception until April 12, 2021. To maximise sensitivity, we also searched for citations in Google Scholar, Scopus, and Web of Science. Search terms included “COVID-19”, “coronavirus”, “inflammation”, “inflammatory storm”, “cytokine”, “cytokine storm”, “anti-inflammatory agents”, and “colchicine”, either separately or in combination. The inhaled steroid budesonide was shown in the open-label STOIC trial to reduce the requirement for urgent medical care in patients with early COVID-19. Activation of the NLRP3 inflammasome by SARS-CoV-2 was observed in lung tissues of patients with COVID-19. Prevention of COVID-19 complications in an outpatient setting ideally requires a clinically approved, orally administered, and inexpensive medication targeting the inflammasome with a known favourable safety profile.

**Added value of this study**

Potential clinical benefits of colchicine have been reported in observational studies and two small RCTs (including GRECCO) of patients admitted to hospital with COVID-19. In our COLCORONA double-blinded, placebo-controlled, randomised trial of 4488 non-hospitalised patients, including the 327 (7%) without a mandatory diagnostic test, the effect of colchicine on COVID-19-related clinical events was not statistically significant. Among the 4159 (93%) of patients with PCR-confirmed COVID-19, colchicine led to a lower rate of the composite of death or hospital admission than placebo.

**Implications of all the available evidence**

Given the absence of orally administered therapies to prevent COVID-19 complications in community-treated patients, the burden on health-care systems caused by hospital admissions, and the benefit of colchicine in patients with PCR-proven COVID-19, we propose that colchicine is a safe and inexpensive anti-inflammatory agent that could be considered for use in those at risk of complications. Notwithstanding these considerations, replication in other studies of patients who have positive PCR tests and have been treated in the community (such as the PRINCIPLE trial) is recommended. Additional trials such as the AGILE-ACCORD might enrich our therapeutic armamentarium to prevent COVID-19-related complications in ambulatory patients.

Prevention of COVID-19 complications in an outpatient setting ideally requires a clinically available, orally administered, and inexpensive medication targeting the inflammasome with a known favourable safety and tolerability profile. Colchicine is a potent anti-inflammatory agent used to treat gout, viral pericarditis, coronary disease, and familial Mediterranean fever.^11^, ^12^, ^13^, ^14^ Its mechanism of action is through the inhibition of tubulin polymerisation, with effects on the inflammasome, cellular adhesion molecules, and inflammatory chemokines.^15^, ^16^, ^17^ In an experimental model of acute respiratory-distress syndrome, colchicine was shown to reduce inflammatory lung injury and respiratory failure by interfering with leukocyte activation and recruitment.^18^ Substantial clinical benefits of colchicine have also been reported in observational studies and two randomised controlled trials of patients admitted to hospital with COVID-19.^19^, ^20^, ^21^, ^22^

We did the COLchicine CORONAvirus SARS-CoV-2 (COLCORONA) trial in community-treated patients with COVID-19 to establish the effects of colchicine on complications, including hospital admission and death, as well as its safety and tolerability.

## Methods

### Study design

COLCORONA was a phase 3, randomised, double-blind, adaptive, placebo-controlled, multicentre, investigator-initiated trial comparing orally administered colchicine (0·5 mg twice per day for the first 3 days and then once per day for 27 days thereafter) with placebo in a 1:1 ratio. We have described the rationale for the treatment regimen (appendix p 8). COLCORONA was done in six countries (Brazil, Canada, Greece, South Africa, Spain, and the USA) and was led by the Montreal Heart Institute (Montreal, QC, Canada). The trial protocol was designed by the study steering committee. The protocol was approved by the institutional review board at all centres involved in the six countries that participated in the trial (appendix pp 4–7). All study support activities, including project coordination, data management, site monitoring, and statistical oversight and analyses, were done at the Montreal Health Innovations Coordinating Center (MHICC). The trial was overseen by a data-safety monitoring board of independent experts.

### Trial population

Patients were eligible if they were at least 40 years of age, had received a diagnosis of COVID-19 within 24 h of enrolment, were not currently being treated in hospital and not under immediate consideration for hospital treatment or admission, and presented at least one of the following high-risk criteria: age of 70 years or older, obesity (body-mass index of 30 kg/m^2^ or more), diabetes, uncontrolled hypertension (systolic blood pressure ≥150 mm Hg), known respiratory disease, known heart failure, known coronary disease, fever of at least 38·4°C within the last 48 h, dyspnoea at the time of presentation, bicytopenia, pancytopenia, or the combination of high neutrophil and low lymphocyte counts. The diagnosis of COVID-19 was made by local laboratories using PCR testing on a nasopharyngeal swab specimen. Given the restrictions in laboratory testing early in the pandemic, a diagnosis was also accepted as an epidemiological link with a household member who had a positive nasopharyngeal test result for patients with symptoms compatible with COVID-19, or by a clinical algorithm in a symptomatic patient without an obvious alternative cause, as per official guidelines (appendix p 9).^23^ Women were either not of childbearing potential or practicing adequate contraception.

Patients were excluded if they had inflammatory bowel disease or chronic diarrhoea or malabsorption, pre-existent progressive neuromuscular disease, an estimated glomerular filtration rate of less than 30 mL per min per 1·73 m^2^, severe liver disease, current treatment with colchicine, current chemotherapy for cancer, or a history of substantial sensitivity to colchicine. Further details regarding eligibility criteria are provided (appendix).

Written informed consent was obtained electronically or on paper from all patients before enrolment following a telemedicine or in-person visit.

### Randomisation and masking

Masked randomisation was centralised and done electronically through an automated interactive web-response system (IWRS). Participants were randomly assigned (1:1) to either colchicine treatment or placebo, using an allocation sequence that was computer-generated using a blocking schema with block sizes of six. Allocation sequence was not stratified. Eligible patients were randomly assigned by research nurses through the IWRS system that provided the bottle number to send to patients. The randomisation list was computer-generated by an unmasked biostatistician and uploaded to an interactive web response system (Dacima). The database was a validated electronic-data-capture system (eCRF) using InForm 6.0 provided by Oracle. The eCRF was developed by the MHICC as per their internal standard-operating procedures. All eCRF users were trained as per completion guidelines and the data entry was done directly by the study staff during phone calls with the patients. The data cleaning activities were done as per the MHICC data-management plan. All staff involved, including study investigators, nurses, and patients were masked to the treatment received.

### Procedures

All patients received either 0·5 mg colchicine orally administered twice per day for the first 3 days and then once per day for 27 days thereafter, or matching placebo. Study medication was delivered at the patient's house within 4 h of enrolment. The study medication and matching placebo were provided by Pharmascience (Montreal, Canada), which had no role in the design or conduct of the trial or the preparation or review of this manuscript.

Clinical evaluations occurred by telephone at 15 days and 30 days following randomisation for evaluation of the occurrence of any trial endpoints or other adverse events.

### Endpoints

The primary efficacy endpoint was a composite of death or hospital admission because of COVID-19 infection in the 30 days after randomisation. The secondary endpoints consisted of the components of the composite primary endpoint, and the need for mechanical ventilation in the 30 days after randomisation. Pneumonias, other serious adverse events, and non-serious adverse events were also collected.

### Statistical analysis

Assuming a primary endpoint event rate of 7% in the placebo group, we estimated that a sample size of approximately 6000 patients randomly allocated to treatment with 3000 patients in each treatment group would be required to detect a target 25% relative-risk reduction with colchicine (corresponding to a primary endpoint event rate of 5·25% with colchicine, for an absolute difference of 1·75%) with a power of 80% and a two-sided test at the 0·05 significance level. Because the efficacy interim analyses were done with the conservative O'Brien-Fleming approach, their impact on final significance was deemed to be minimal and no sample-size adjustment was done for interim analyses.

Efficacy analyses were done according to the intention-to-treat principle. The primary endpoint was compared between the two treatment groups using a χ^2^ test, and the odds ratio (OR) along with the 95·1% CI was provided. Secondary endpoints were analysed similarly. Because of potential limitations to the specificity of COVID-19 diagnosis made on clinical or epidemiological criteria alone, a pre-specified subgroup analysis of the primary endpoint examined patients who were enrolled based on a positive PCR test. Pre-specified subgroup analyses of the primary endpoint were done using logistic-regression models including the treatment group, the subgroup factor, and the treatment x subgroup-factor interaction. Investigation of secondary endpoints in subgroups were done as post-hoc analyses. A pre-specified sensitivity analysis of the primary endpoint was done by imputing a primary event in event-free patients who did not complete the study (ie, discontinued before day 30 or for whom no information was available at end of study).

Three formal interim efficacy analyses on the primary endpoint were planned after 25%, 50%, and 75% of the primary endpoint events had occurred. The prespecified stopping rule for efficacy was based on the Lan-DeMets procedure with the O'Brien-Fleming α-spending function to determine the significance level. Futility was assessed by computing the conditional power under the original alternative and judged at 15%. Results of the interim analyses were generated by an unmasked biostatistician and were provided only to the data-safety monitoring board members. During the entire duration of the trial, the study team, including the biostatisticians who wrote the statistical analysis plan and generated the final results, remained masked to treatment allocation.

Following its review of the first two interim results, the monitoring board recommended that the trial should continue as planned (appendix p 10). On Dec 11, 2020, the steering committee chairman informed the data-safety monitoring board that the investigators had decided to terminate the study once 75% of the planned patients were recruited and had completed the 30 day follow-up. This decision was made due to substantial logistical, personnel, and budgetary issues related to maintaining the central study call centre active 24 h per day for a prolonged period of time, which were compounded by the inability to reliably model the additional time required to reach the target number of patients through the successive waves of the pandemic. To account for the two interim analyses that were done, the final statistical significance level was calculated as 0·049 for the final analysis of the primary endpoint. No other statistical adjustment for bias was made. All other statistical tests were two-sided and done at the 0·05 significance level.

Statistical analyses were done using SAS version 9.4. There was no prespecified plan to adjust for multiple comparisons across the multiple methods used to analyse the primary outcome and secondary endpoints; results of these analyses are reported with point estimates and 95% CI, without p values. 95% CIs are not adjusted for multiple comparisons, and inferences drawn from them might not be reproducible. The statistical analysis plan was approved on Nov 25, 2020. The COLCORONA trial is registered with ClinicalTrials.gov (NCT04322682).

### Role of the funding source

The funder of the study had no role in the study design, data collection, data analysis, data interpretation, or writing of the report.

## Results

Trial enrolment began in March 23, 2020, and was completed in Dec 22, 2020; the last trial visit was in Jan 19, 2021. A total of 4488 patients were randomly assigned to either colchicine (2235 patients) or placebo (2253 patients) and were followed up for 30 days. At the time of database lock and unblinding on Jan 20, 2021, vital and primary-endpoint event status were available for all except 93 (2·1%) of 4488 patients (figure).

**Figure:**
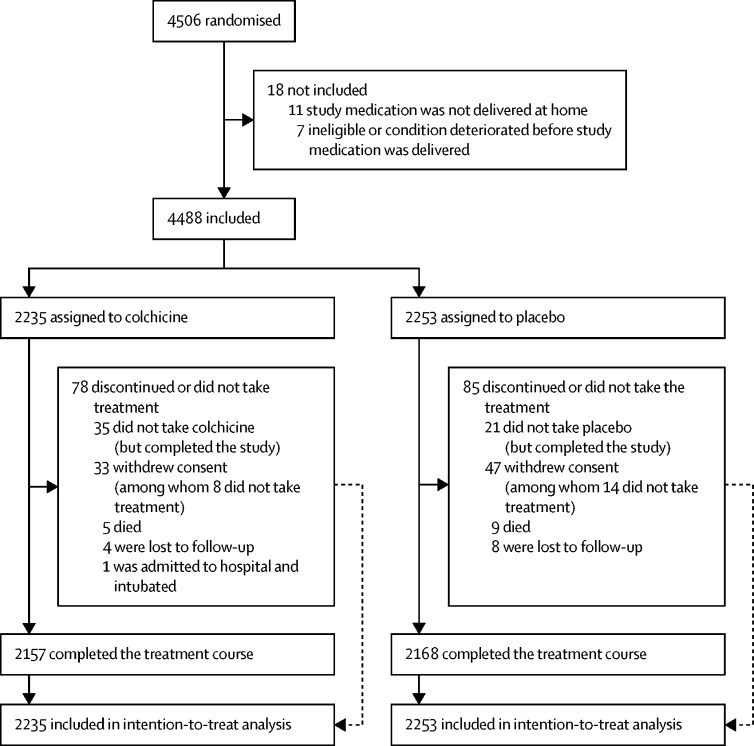
Trial profile Of a sample of 5536 patients screened in Canada, 775 (14%) patients were randomly assigned to treatment, because 2392 (43%) patients did not have at least one high-risk characteristic (which was an inclusion criterion) or met at least one exclusion criterion, and 2369 (43%) patients declined participation. Note that information on screened individuals is only available for those recruited in Canada.

We present the baseline characteristics of the patients (table 1, appendix p 12). Patients were enrolled a mean of 5·3 days (SD 4·4) after the onset of COVID-19 symptoms. The median age of participants was 54·0 years (IQR 47·0–61·0), 53·9% of the patients were women, mean body-mass index was 30·0 kg per m^2^ (SD 6·2), and 19·9% had diabetes. The mean treatment duration with the trial medication was 26·2 days (SD 9·6).

**Table 1:** Characteristics of the patients at randomisation in the intent-to-treat population

		**Colchicine (n=2235)**	**Placebo (n=2253)**
Age (years), median (IQR)		53·0 (47·0–61·0)	54·0 (47·0–61·0)
Women		1238 (55·4%)	1183 (52·5%)
Men		997 (44·6%)	1070 (47·5%)
White*		2086 (93·3%)	2096 (93·2%)
Black		114 (5·1%)	119 (5·3%)
BMI (kg/m^2^), mean (SD)		30·0 (6·2)	30·0 (6·3)
Smoking		217 (9·7%)	212 (9·4%)
Hypertension		781 (34·9%)	848 (37·6%)
Diabetes		444 (19·9%)	450 (20·0%)
Respiratory disease		583 (26·1%)	605 (26·9%)
Prior MI		65 (2·9%)	72 (3·2%)
Prior heart failure		24 (1·1%)	18 (0·8%)
Concomitant medication use			
	Hydroxychloroquine	12 (0·5%)	11 (0·5%)
	Oral anticoagulant	48 (2·1%)	65 (2·9%)
	Aspirin	195 (8·7%)	235 (10·4%)
	Other platelet agents	32 (1·4%)	43 (1·9%)
Countries			
	Canada	1817 (81·3%)	1830 (81·2%)
	USA	244 (10·9%)	244 (10·8%)
	Other countries	174 (7·8%)	179 (7·9%)

Data are n (%) unless otherwise specified. BMI=body-mass index. MI=myocardial infarction.

* Data on race were missing for some patients; the denominator in the placebo group was 2248.

A primary endpoint event occurred in 104 (4·7%) of 2235 patients in the colchicine group, as compared with 131 (5·8%) of 2253 patients in the placebo group (table 2). Components of the primary endpoint included death and hospital admission due to COVID-19, and for the secondary efficacy endpoint included the need for mechanical ventilation. Although not a prespecified endpoint, the rate of hospital admissions due to any cause was 110 (4·9%) of 2235 patients in the colchicine group and 138 (6·1%) of 2253 patients in the placebo group (OR 0·79, 95% CI 0·61–1·03; p=0·078).

**Table 2:** Rates and odds ratios for major clinical outcomes in the intent-to-treat population

		**Colchicine (n=2235)**	**Placebo (n=2253)**	Odds ratio (95% CI)*	**p value**
Primary composite endpoint		104 (4·7%)	131 (5·8%)	0·79 (0·61–1·03)	0·081
Components of primary endpoint					
	Death	5 (0·2%)	9 (0·4%)	0·56 (0·19–1·67)	..
	Hospitalisation for COVID-19	101 (4·5%)	128 (5·7%)	0·79 (0·60–1·03)	..
Secondary endpoint, mechanical ventilation		11 (0·5%)	21 (0·9%)	0·53 (0·25–1·09)	..

Data are n (%).

* Odds ratio (95·1% CI) for the primary composite endpoint.

In a prespecified sensitivity analysis of the primary endpoint to account for missing data where an event was imputed in all patients who were event free and who did not complete the study (32 patients in the colchicine group and 49 patients in the placebo group), the primary-endpoint event rate was 136 (6·1%) of 2235 patients in the colchicine group and 180 (8·0%) of 2253 patients in the placebo group (OR 0·75, 95% CI, 0·59–0·94; p=0·013).

In the prespecified analysis of the 4159 patients with COVID-19 confirmed by PCR (appendix p 13), the primary endpoint was shown in 96 (4·6%) of 2075 patients in the colchicine group and 126 (6·0%) of 2084 in the placebo group (table 3). Among the patients with confirmed COVID-19, ORs were 0·75 (95% CI 0·57–0·99) for hospital admission due to the infection and 0·56 (0·19–1·66) for death. Results for the secondary endpoints in this group of patients represented a post-hoc analysis. The secondary efficacy endpoint of the need for mechanical ventilation occurred in ten (0·5%) of 2075 patients in the colchicine group, as compared with 20 (1·0%) of 2084 patients in the placebo group (table 3). Although not a prespecified endpoint, the rate of hospital admission due to any cause was 101 (4·9%) of 2075 patients in the colchicine group versus 132 (6·3%) of 2084 patients in the placebo group (OR 0·76, 95% CI 0·58–0·99; p=0·040).

**Table 3:** Rates and odds ratios for major clinical outcomes in the subgroup of patients with PCR-confirmed COVID-19 in the intent-to-treat population

		**Colchicine (n=2075)**	**Placebo (n=2084)**	**Odds ratio (95% CI)**	**p value**
Primary composite endpoint		96 (4·6%)	126 (6·0%)	0·75 (0·57–0·99)	0·042
Components of primary endpoint					
	Death	5 (0·2%)	9 (0·4%)	0·56 (0·19–1·66)	..
	Hospitalisation for COVID-19	93 (4·5%)	123 (5·9%)	0·75 (0·57–0·99)	..
Secondary endpoint mechanical ventilation		10 (0·5%)	20 (1·0%)	0·50 (0·23–1·07)	..

Data are n (%). Evaluation of the primary endpoint in the subgroup of patients with PCR-confirmed COVID-19 was prespecified and that of components of the primary endpoint and the secondary endpoints were done as post-hoc analyses.

Among the patients with diabetes, the primary endpoint occurred in 27 (6·1%) of the 444 patients in the colchicine group and 43 (9·6%) of the 450 in the placebo group. The primary endpoint event rate in men was 58 (5·8%) of 997 in the colchicine group and 90 (8·4%) of 1070 in the placebo group, whereas the corresponding values in women were 46 (3·7%) of 1238 in the colchicine group and 41 (3·5%) of 1183 in the placebo group (table 4). The primary-event rate in the placebo group was 126 (6·0%) of 2084 when diagnosis was done by PCR, and five (3·0%) of 169 when clinical criteria were used for diagnosis.

**Table 4:** Primary-efficacy composite endpoint in prespecified subgroups of the intent-to-treat population

	**Colchicine**	**Placebo**	**Odds ratio (95% CI)**
**PCR-confirmed COVID-19**			
Yes	96 (4·6%) of 2075	126 (6·0%) of 2084	0·75 (0·57–0·99)
No	8 (5·1%) of 158	5 (3·0%) of 169	1·75 (0·56–5·46)
**History of diabetes**			
Yes	27 (6·1%) of 444	43 (9·6%) of 450	0·61 (0·37–1·01)
No	77 (4·3%) of 1791	88 (4·9%) of 1803	0·88 (0·64–1·20)
**History of hypertension**			
Yes	48 (6·1%) of 781	64 (7·5%) of 848	0·80 (0·54–1·18)
No	56 (3·9%) of 1454	67 (4·8%) of 1405	0·80 (0·56–1·15)
**Smoking**			
Non-smoker	59 (4·6%) of 1279	71 (5·6%) of 1270	0·82 (0·57–1·16)
Previous smoker	38 (5·1%) of 738	56 (7·3%) of 770	0·69 (0·45–1·06)
Active smoker	7 (3·2%) of 217	4 (1·9%) of 212	1·73 (0·50–6·01)
**Age**			
≥70 years	18 (9·5%) of 190	27 (12·7%) of 213	0·72 (0·38–1·36)
<70 years	86 (4·2%) of 2045	104 (5·1%) of 2040	0·82 (0·61–1·09)
**Sex**			
Men	58 (5·8%) of 997	90 (8·4%) of 1070	0·67 (0·48–0·95)
Women	46 (3·7%) of 1238	41 (3·5%) of 1183	1·07 (0·70–1·65)
**Race**			
Black	3 (2·6%) of 114	6 (5·0%) of 119	0·51 (0·12–2·09)
Non-black	101 (4·8%) of 2121	125 (5·9%) of 2129	0·80 (0·61–1·05)
**Body-mass index**			
≥30 kg/m^2^	53 (5·2%) of 1012	70 (6·7%) of 1040	0·77 (0·53–1·11)
<30 kg/m^2^	50 (4·1%) of 1216	61 (5·1%) of 1205	0·80 (0·55–1·18)
**Respiratory disease**			
Yes	35 (6·0%) of 583	48 (7·9%) of 605	0·74 (0·47–1·16)
No	69 (4·2%) of 1652	83 (5·0%) of 1647	0·82 (0·59–1·14)
**Cardiovascular disease**			
Yes	6 (5·0%) of 119	11 (9·0%) of 122	0·54 (0·19–1·50)
No	98 (4·6%) of 2116	120 (5·6%) of 2131	0·81 (0·62–1·07)
**Use of ACEi or ARB**			
Yes	37 (6·1%) of 602	53 (7·8%) of 676	0·77 (0·50–1·19)
No	67 (4·1%) of 1633	78 (4·9%) of 1577	0·82 (0·59–1·15)

Data are n (%) of N. ACEi=angiotensin-converting enzyme inhibitor. ARB=angiotensin-receptor blocker.

Serious adverse events occurred in 4·9% in the colchicine group and 6·3% in the placebo group (table 5), and pneumonia occurred in 2·9% in the colchicine group and 4·1% in the placebo group. Pulmonary embolism was diagnosed in 0·5% of patients in the colchicine group and 0·1% in the placebo group (appendix p 14). These pulmonary emboli did not lead to the need for mechanical ventilation or to death, were all considered to be unrelated to the study medication by the physician in charge, and were included as hospital admissions due to COVID-19 in the analysis of the primary efficacy endpoint. No deep venous thrombosis was reported. Dehydration was reported in three (0·1%) of 2195 patients in the colchicine group and six (0·3%) of 2217 patients in the placebo group.

**Table 5:** Proportions of patients with adverse events in the safety population

	**Colchicine (n=2195)**	**Placebo (n=2217)**
Death	5 (0·2%)	9 (0·4%)
Any SAE	108 (4·9%)	139 (6·3%)
Pneumonia SAE	63 (2·9%)	92 (4·1%)
Pulmonary embolism	11 (0·5%)	2 (0·1%)
Deep venous thrombosis	0	0
Myocardial infarction	0	1 (<0·1%)
Dehydration SAE	3 (0·1%)	6 (0·3%)
Any related AE	532 (24·2%)	344 (15·5%)
Gastrointestinal AE	524 (23·9%)	328 (14·8%)
Gastrointestinal SAE	6 (0·3%)	3 (0·1%)
Diarrhoea AE	300 (13·7%)	161 (7·3%)
Nausea AE	43 (2·0%)	47 (2·1%)
Gastrointestinal haemorrhage AE	1 (<0·1%)	0
Rash AE	4 (0·2%)	13 (0·6%)

Data are n (%). The safety population refers to the patients who took at least one dose of trial medication. AE=adverse event. SAE=serious adverse event. NA=not applicable.

The percentage of adverse events that were considered to be related to trial medication was 24·2% in the colchicine group and 15·5% in the placebo group (table 5). At least one treatment-emergent gastrointestinal adverse event occurred in 524 (23·9%) of 2195 patients in the colchicine group, as compared with 328 (14·8%) of the 2217 patients in the placebo group. Diarrhoea was reported in 300 (13·7%) of 2195 patients in the colchicine group and 161 (7·3%) of 2217 patients in the placebo group.

## Discussion

In COLCORONA, the risk of the primary composite efficacy endpoint of death or hospital admission due to COVID-19 infection in the 30 days following randomisation was not statistically significantly lower among the patients who were randomly assigned to receive colchicine than in those who received placebo.

Because of the shortage of reagents for PCR tests and the restriction in the use of such testing early in the pandemic, diagnosis of probable COVID-19 through an epidemiological link or compatible symptoms was initially allowed in the study. These patients had a primary event rate that was half (3%) that observed in those with confirmed diagnosis by PCR testing (6%).

When the patients who had a confirmed diagnosis of COVID-19 are considered, the benefit of colchicine on the primary-efficacy endpoint was more marked and statistically significant. The relative-risk reduction that we observed was similar to the one planned in the sample-size calculation. Treatment with colchicine was associated with concordant effects on hospital admissions, use of mechanical ventilation, and deaths in patients with a diagnosis of COVID-19 confirmed by PCR testing.

The effect of colchicine on the primary endpoint was consistent across subgroups of patients on the basis of various clinical characteristics. Although the benefits of colchicine appeared to be more marked in patients with diabetes and men, there was no statistically significant heterogeneity in the results. Because the event rates were higher in patients with these characteristics, the effect of colchicine might have been more readily detectable. Diabetes is a pro-inflammatory state, which might explain the greater risk of complications of COVID-19 in patients with diabetes than in those without. Despite the link between weight, insulin resistance, and type 2 diabetes, the effects of colchicine did not differ whether the body-mass index was higher or lower than 30 kg per m^2^. Sex-related differences in immune responses against SARS-CoV-2 exist. Men have higher plasma concentrations of IL-18 and IL-8, whereas women have stronger T-cell activation.^24^ These differences might at least in part explain the apparent difference in response to colchicine in COVID-19. Of note, the concomitant use of an inhibitor of the renin-angiotensin system did not appear to modify the clinical response to colchicine.

The most common adverse events that we observed were gastrointestinal. Diarrhoea was reported by 13·7% of patients in the colchicine group and 7·3% in the placebo group. Dehydration was reported in three (0·1%) patients in the colchicine group and six (0·3%) patients in the placebo group. Deleterious effects of COVID-19 are numerous and can affect among others the lungs, heart, and brain. The seriousness of the disease is reflected by the high incidence of serious adverse events in patients of the placebo group in the COLCORONA trial. The number of patients with any serious adverse event was smaller in the colchicine group than in the placebo group, which might reflect the benefits of systemic-inflammation reduction in this disease. Pneumonia was reported less frequently in patients of the colchicine group than those of the placebo group. This is concordant with the statistically significant reduction of hospital admissions in patients with confirmed COVID-19 treated with colchicine, and the numerically lower number of patients requiring mechanical ventilation, although this was not significant. Our results are supported by the results of two smaller clinical trials showing reductions in the need for oxygen supplementation, the duration of hospital treatment, and the deterioration of clinical status.^19^, ^20^ Colchicine has previously been shown to reduce acute lung injury in an experimental model of acute respiratory-distress syndrome.^18^ The risk of viral inflammatory pneumonitis therefore appears to be lowered by colchicine in patients with COVID-19. Reassuringly, there was no evidence of an increased risk of bacterial pneumonia in COLCORONA.

The number of reported cases of pulmonary embolism was higher in patients of the colchicine group than in patients in the placebo group in COLCORONA. The 13 cases of pulmonary embolism were included in the analysis of the primary composite endpoint as hospital admissions due to COVID-19. Despite this apparent imbalance, the numbers of hospital admissions, use of mechanical ventilation, and deaths were numerically lower in the colchicine group than in the placebo group. Additionally, the pulmonary emboli did not lead to the need for mechanical ventilation or to death in these 13 patients. By contrast, there was no imbalance in other reported thrombotic or embolic events such as deep venous thrombosis or myocardial infarction. A systematic review and meta-analysis of randomised controlled trials involving more than 10 000 patients with coronary disease followed for approximately 2 years has not shown a statistically significant difference in the rate of pulmonary embolism or deep venous thrombosis between the colchicine and placebo groups.^25^ In addition, a significant reduction in the concentration of D dimer, a plasma biomarker used for the diagnostic investigation of pulmonary embolism, was observed with colchicine compared with the control in the GRECCO study.^19^ Furthermore, colchicine has previously been shown in murine models to lower the release of α defensin associated with large thrombus burdens and in clinical studies to reduce the aggregation between neutrophils and platelets.^26^, ^27^, ^28^ Thus, there is no known potential biological basis to suggest a causal link between colchicine therapy and thromboembolic disease. The incidence of pulmonary embolism is high in COVID-19, and occurs in up to 25% of patients with associated pneumonia.^29^ More than 57% of patients dying from COVID-19 have evidence of deep venous thrombosis or pulmonary embolus at autopsy.^30^ In COLCORONA, the incidence of pulmonary embolism in patients requiring hospital admission was 11 (10·9%) of 101 patients in the colchicine group and two (1·6%) of 128 in the placebo group. The totality of the evidence suggests that the low rate of pulmonary embolisms in the placebo group represents an anomaly. The more common presence and greater severity of COVID-19 pneumonia infiltrates in patients receiving placebo might have reduced the clinical suspicion for pulmonary embolism in symptomatic patients.

Our trial has certain limitations. The study was stopped when 75% of the planned patients were recruited and had completed the 30 day follow-up. We nevertheless believe that our results are clinically persuasive. The duration of follow-up was relatively short at approximately 30 days. We did not investigate the evolution of persistent COVID-19 symptoms and the effects of longer-term treatment with colchicine. The benefit of a shorter course of colchicine therapy for less than 30 days is also not entirely known, although the results of two smaller trials showed benefits of treatment administered for 10–21 days.^19^, ^20^ Finally, our results apply to patients who have a proven diagnosis of COVID-19, are at risk of clinical complications, and are not admitted to hospital at the time of treatment initiation. The number needed to treat with colchicine to prevent one primary endpoint event was 70 (95% CI 36–1842) for patients with PCR-proven COVID-19, 29 (14 to not defined) for those with diabetes, 31 (11 to not defined) for patients aged 70 years and older, 39 (21–259) in men, 52 (21 to not defined) in patients with respiratory disease, and 25 (10 to not defined) in those with cardiovascular disease.

In conclusion, in community-treated patients including those without a mandatory diagnostic test, the effect of colchicine on COVID-19-related clinical events was not statistically significant. Among patients with PCR-confirmed COVID-19, colchicine led to a lower rate of the composite of death or hospital admission than placebo.

## Data sharing

The anonymised patient data will be shared, while safeguarding the privacy of participants, via the Vivli data repository (https://vivli.org/).
