# Supplementary material for: Colchicine for community-treated patients with COVID-19 (COLCORONA): a phase 3, randomised, double-blinded, adaptive, placebo-controlled, multicentre trial
Source: Lancet Respir Med. 2021 Aug;9(8):924–32. doi: 10.1016/S2213-2600(21)00222-8 (PMC8159193; doi:10.1016/S2213-2600(21)00222-8)
Supplement: Supplementary appendix [file mmc1.pdf]

THE LANCET
Respiratory Medicine

Supplementary appendix

This appendix formed part of the original submission and has been peer reviewed. We post it as supplied by the authors.

Supplement to: Tardif J-C, Bouabdallaoui N, L'Allier PL, et al. Colchicine for community-treated patients with COVID-19 (COLCORONA): a phase 3, randomised, double-blinded, adaptive, placebo-controlled, multicentre trial. *Lancet Respir Med* 2021; published online May 27. http://dx.doi.org/10.1016/S2213-2600(21)00222-8.

Colchicine for community-treated patients with COVID-19 (COLCORONA): A phase 3, randomised, double-blinded, adaptive, placebo-controlled multicentre trial

Supplementary materials

Table of contents

COLCORONA – Steering Committee members 2
COLCORONA - Data Safety Monitoring Board members 3
COLCORONA - Full list of trial investigators with their institutional affiliations 4
Supplementary Tables 8
Table S1 – Rationale for the colchicine treatment regimen 8
Table S2 – Diagnosis of COVID-19 in COLCORONA 9
Table S3 – Results of the two formal interim analyses by the data safety monitoring board 10
Table S4 Prevalence of high-risk criteria leading to inclusion of patients in the trial for a sample of 775 patients recruited in Canada[a] 12
Table S5 Characteristics of the patients at randomization in the prespecified subgroup of patients with PCR-proven COVID-19 in the intent-to-treat population 13
Table S6 Patients with pulmonary embolism 14

COLCORONA – Steering Committee members

Chair: **Jean-Claude Tardif,** MD, Montreal Heart Institute, Université de Montréal, Montreal, Canada

Guy Boivin, MD, MSc, Centre Hospitalier Universitaire de Québec and Université Laval, Quebec City, Canada; **Nadia Bouabdallaoui**, MD, PhD, Montreal Heart Institute, Université de Montréal, Montreal, Canada; **Protasion da Luz**, MD, MBA, Instituto do Coração, Hospital das Clínicas, Faculdade de Medicina, Universidade de São Paulo, São Paulo, Brasil; **André Denault**, MD, PhD, Montreal Heart Institute, Université de Montréal, Montreal, Canada; **Marie-Pierre Dubé**, PhD, Université de Montréal Pharmacogenomics Center, Montreal Heart Institute, Montreal, Canada; **Jocelyn Dupuis**, MD, PhD, Montreal Heart Institute, Université de Montréal, Montreal, Canada; **Daniel Gaudet**, MD, PhD, Ecogene-21 and Department of Medicine, Université de Montréal, Chicoutimi, Canada; **Philippe L. L'Allier**, MD. Montreal Heart Institute, Université de Montréal, Montreal, Canada; **Jose Lopez-Sendon**, MD, Cardiology department Hospital Universitario La Paz. UAM. IdiPaz. CIBER-CV, Madrid, Spain; **Martin Pelletier**, PhD, Centre Hospitalier Universitaire de Québec and Université Laval, Quebec City, Canada; **Michael H. Pillinger**, MD, New York University Grossman School of Medicine, New York; **Binita Shah**, MD, New York University Grossman School of Medicine, New York; **Philippe A. Tessier**, PhD, Centre Hospitalier Universitaire de Québec and Université Laval, Quebec City, Canada; **David D. Waters**, MD, Division of Cardiology, Zuckerberg San Francisco General Hospital, Department of Medicine, University of California, San Francisco.

COLCORONA - Data Safety Monitoring Board members

Chair: **Jean-Lucien Rouleau**, MD, Université de Montréal, Montreal, Canada

Marc Pfeffer, MD, PhD, Brigham and Women's Hospital, Harvard Medical School, Boston; **Lisa Rosenbaum**, MD, Brigham and Women's Hospital, Harvard Medical School; **Cécile Tremblay,** MD, Centre Hospitalier Universitaire de Montréal, Université de Montréal, Montreal, Canada; **Georges Wells**, PhD, University of Ottawa Heart Institute, Ottawa, Canada; **George Wyse**, MD, Libin Cardiovascular Institute, University of Calgary, Alberta, Canada.

COLCORONA - Full list of trial investigators with their institutional affiliations

BRAZIL

**PI name**	**Site name**	**City**
Antunes, M.	Hospital Universitário Bragança Paulista	Sao Paulo
Chaves E.B.M.	Hospital de Clinicas de Porto Alegre	Porto Alegre
da Luz, P.L.	Instituto do Coração HCFMUSP	Sao Paulo
Mattos C.	Hospital de Clínicas de Passo Fundo	Passo Fundo
Viecili, P.R.N.	Instituto Cruzaltense de Cardiologia - Health Interdisciplinary Research Laboratory Group	Centro Cruz Alta
Ripardo, J.P.	Hospital Samaritano Higienópolis	Sao Paulo

CANADA

**PI name**	**Site name**	**City**
Azzari, F. A.	CISSS Bas Saint-Laurent – Hôpital Régional de Rimouski	Rimouski
Bérubé, S.	CIUSSS de l'Estrie – Centre Hospitalier Universitaire de Sherbrooke	Sherbrooke
Bouabdallaoui, N.	Montreal Heart Institute	Montreal
Conway, B.	Vancouver Infectious Diseases Research and Care Centre Society	Vancouver
Deb, S.	Sunnybrook Health Sciences Center (on Ontario ICF)	Toronto
Francoeur, B.	CIC Mauricie	Trois-Rivières
Fremes, SE.	Sunnybrook Health Sciences Center (on Ontario ICF)	Toronto
Gaudet, D.	Ecogene-21	Chicoutimi
Khoury, E.	Ecogene-21	Chicoutimi
Lavoie-L'Allier, P.	Montreal Heart Institute	Montreal
Tardif, JC	Montreal Heart Institute	Montreal

GREECE

PI name	Site name	City
Deftereos, S.	University General Hospital of Athens "Attikon"	Athens
Randou, E.	General Hospital of Kozani "Mamatsio"	Kozani

SOUTH AFRICA

PI name	Site name	City
Prozesky, H.	Tread Research, Tygerberg Hospital	Cape Town

SPAIN

PI name	Site name	City
Alfonso, F.	Hospital Universitario de La Princesa	Madrid
Arribas, F.	Hospital Universitario 12 de Octubre	Madrid
Calvin, M.E.	Hospital Universitario La Paz	Madrid
Del Val, D.	Hospital Universitario de La Princesa	Madrid
Garcia Villa, A.	Fundación Jiménez Díaz	Madrid
Lopez-Sendon, J.	Hospital Universitario La Paz	Madrid
Lopez-Sendon Moreno, J.	Hospital Universitario Ramón y Cajal	Madrid
Martin-Jiminez, ML.	Hospital Universitario Puerta de Hierro	Madrid
Munoz, P.	Hospital General Universitario Gregorio Marañón	Madrid
Perez Torre, P.	Hospital Universitario Ramón y Cajal	Madrid
Segovia, J.	Hospital Universitario Puerta de Hierro	Madrid
Tunon, J.	Fundación Jiménez Díaz	Madrid

UNITED STATES

PI name	Site name	City
Al Rabadi, O.	Yuma Regional Medical Center	Yuma
Baker, WF.	Central Cardiology Medical Center	Bakersfield
Berney, S.	University of Arkansas for Medical Sciences, Institute on Aging	Little Rock
Brodetskiy, K.	Woodhull Hospital	Brooklyn
Costanzo, J.	Stamford Health	Stamford
Hoang, TA.	Spring Clinical Research	Spring, TX
Hsue, P.	Zuckerberg San Francisco General Hospital	San Francisco
Joshi, A.	Mayo Clinic – Rochester	Rochester
Kennish, L.	Summit Medical Group	New Jersey
King, C.M.	North Mississsippi Medical Clinic	Tupelo
Kole A.	Mayo Clinic - Phoenix	Phoenix
Leibowitz, E.	Valley Health	Ridgewood
Lepor, N.	Westside Medical Associates of Los Angeles	Beverley Hills
Makam, S.	Newburgh Horizon/Mid Hudson Medical	New York
Matejicka, A.	Montefiore Nyack Hospital	New York
Meisner, J.	University of Texas Southwestern Medical Center	Dallas
Nader, R.	Miami Center for Advanced Cardiology	Miami Beach
Orenstein, R.	Mayo Clinic - Phoenix	Phoenix
Pillinger, M.H.	NYU Grossman School of Medicine	New York
Radford, M.	NYC Health and Hospitals, Hackensack Meridian Health	New York
Raiszadeh, F.	Harlem Hospital	New York
Rank, M.	Mayo Clinic - Phoenix	Phoenix
Rollins, N.	University of Texas Southwestern Medical Center	Dallas
Schnee, A.	Prisma Health - Upstate	Greenville

PI name	Site name	City
Shah, B.	NYU Grossman School of Medicine	New York
Shaw, S.	Rancho Research Institute at Rancho Los Amigos National Rehabilitation Center	Downey
Sheikh, SZ.	University of North Carolina at Chapel Hill	Chapel Hill
Shue, P.	University of California San Francisco	San Francisco
Speicher, L.	Mayo Clinic - Jacksonville	Jacksonville
Szerlip, HM.	Baylor Scott & White Research Institute	Dallas
Yang, F.	Maimonides Hospital	New York
Yanez, G.	South Florida Research Organization	Miami

Supplementary Tables

Table S1 – Rationale for the colchicine treatment regimen

The dose of 0·5 mg of colchicine used in COLCORONA was the one utilized in the COLCOT (Tardif JC et al, N Engl J Med 2019;381:2497-2505) and LoDoCo2 (SM Nidorf et al, N Engl J Med 2020;383:1831-47) trials. The posology (colchicine 0·5 mg twice daily for the first 3 days and then once daily for 27 days) used in COLCORONA was based on the following rationale : a) colchicine 0·5 mg once daily was efficacious in the COLCOT and LoDoCo2 trials in more than 10,000 patients with coronary artery disease; b) colchicine 0·5 mg once daily has been shown to have anti-inflammatory effects; c) colchicine 0·5 mg twice daily is given for patients weighing 70 kg or more in acute pericarditis; d) colchicine causes more diarrhea when 0·5 mg twice daily is administered (compared to 0·5 mg once daily); e) COVID-19 can also cause diarrhea; f) the combination of colchicine-related and COVID-related diarrhea might affect compliance to the study medication; g) the hypothesis underlying COLCORONA was that rapid prevention or control of the inflammatory storm is highly desirable to prevent related complications; h) the evolution of COVID-19 was not perfectly characterized at the time of study launch in March 2020 but the treatment duration of 30 days would entirely cover the period during which patients are at risk of short-term complications (the long-hauler issue was not known at study launch); i) the posology used in COLCORONA (colchicine 0·5 mg twice daily for the first 3 days and then once daily for 27 days) allowed the obtaining of adequate colchicine levels more rapidly (hence the 0·5 mg twice daily posology for the first 3 days) while both maintaining adequate anti-inflammasome inhibition and limiting gastro-intestinal side effects for the full treatment period (hence the 0·5 mg once daily poslogy for the following 27 days).

Table S2 – Diagnosis of COVID-19 in COLCORONA

1- Confirmed diagnosis by naso-pharyngeal swab and COVID-19 PCR test, or

2- Clinical diagnosis by epidemiological link with COVID-19-compatible symptoms and member of household with positive nasopharyngeal COVID-19 PCR test result, or

3- Clinical diagnosis of probable COVID-19 according to criteria adapted from official guidelines, in patients with sudden onset of the following symptoms without an obvious alternative cause:

3a- Fever (> 38 degrees C) and cough or

3b- Fever (> 38 degrees C) or cough with at least one of the following symptoms:

- shortness of breath
- extreme fatigue
- muscle or joint pains
- sudden anosmia without nasal obstruction, with or without agueusia

Table S3 – Results of the two formal interim analyses by the data safety monitoring board

The stopping boundaries for efficacy were computed using the Lan-DeMets procedure with the O'Brien-Fleming alpha spending function and are presented in the maximum likelihood estimate (MLE) scale (which corresponds to the difference between the primary event rate of each group) and the p-value scale for the two interim analyses.

Stopping boundaries for efficacy based on observed data

	**Observed results for primary endpoint**	**Lower boundary (MLE scale)**	**Upper boundary (MLE scale)**	**Boundary (p-value scale)**
**First Interim analysis**	Placebo: 56/822 (6·8%)<br>Colchicine: 39/822 (4·7%)<br>MLE = 0·0207<br>p-value = 0·0724	-0·04604	0·04604	0·000062
**Second Interim analysis**	Placebo: 107/1860 (5·8%)<br>Colchicine: 83/1847 (4·5%)<br>MLE = 0·0126<br>p-value = 0·0822	-0·01713	0·01713	0·017382

At both interim analyses, the observed results did not cross the stopping boundaries so based on these statistical stopping rules, the study was not stopped for efficacy.

The stopping rule for futility was based on the conditional power that was computed under the original alternative (25% reduction in event rate) and under the current trend.

Conditional power under various alternative hypotheses

	**Alternative hypothesis (difference in event rates MLE)**	**Conditional Power**
**First Interim analysis**	Original : 0·0175 (25% reduction)	88·3%
	Current trend : 0·0207 (30·4% reduction)	94·7%
**Second Interim analysis**	Original : 0·0175 (25% reduction)	67·4%
	Current trend: 0·0126 (17·9% reduction)	51·5%

The conditional powers were to be compared to a 15% threshold so based on these statistical stopping rules, the study was not stopped for futility.

Table S4 Prevalence of high-risk criteria leading to inclusion of patients in the trial for a sample of 775 patients recruited in Canada[a]

**Characteristic**	**Number of patients (%)**
Dyspnea at presentation	371 (48%)
Obesity (body-mass index ≥ 30 kg/m^2)	320 (41%)
Fever ≥38·4°C within last 48 hours	185 (24%)
Known respiratory disease	146 (19%)
Diabetes	108 (14%)
Hypertension and SBP ≥150 mm Hg	71 (9%)
Age ≥ 70 years	56 (7%)
Know coronary disease	27 (3%)
Known heart failure	3 (0·4%)

Table S5 Characteristics of the patients at randomization in the prespecified subgroup of patients with PCR-proven COVID-19 in the intent-to-treat population

**Characteristic**	**Colchicine (N=2075)**	**Placebo (N=2084)**
Age - years	54·5±9·7	54·9±9·9
Female sex - no. (%)	1167 (56·2%)	1107 (53·1%)
Caucasian - no. (%)	1931 (93·1%)	1938 (93·2%)
Black - no. (%)	111 (5·3%)	111 (5·3%)
Body-mass index (kg/m^2)	30·0±6·3	30·1±6·3
Smoking - no. (%)	194 (9·3%)	188 (9·0%)
Hypertension - no. (%)	724 (34·9%)	789 (37·9%)
Diabetes - no. (%)	408 (19·7%)	421 (20·2%)
Respiratory disease - no. (%)	546 (26·3%)	568 (27·3%)
Prior MI - no. (%)	61 (2·9%)	70 (3·4%)
Prior heart failure - no. (%)	24 (1·2%)	16 (0·8%)
Hydroxychloroquine use - no.(%)	11 (0·5%)	11 (0·5%)
Oral anticoagulant use - no. (%)	45 (2·2%)	64 (3·1%)
Aspirin use - no. (%)	183 (8·8%)	217 (10·4%)
Other antiplatelet agent - no. (%)	27 (1·3%)	38 (1·8%)
Canada - no. (%)	1710 (82·4%)	1717 (82·4%)
United States - no. (%)	225 (10·8%)	225 (10·8%)
Other countries - no. (%)	140 (6·7%)	142 (6·8%)

Abbreviation: MI, myocardial infarction.

Table S6 Patients with pulmonary embolism

Patient	Study arm	Sex	Age (years)	PE proximal to subsegment	Day of[a] onset	Presence of pneumonia	Relation to study med	PE leading to MV	Death
**1**	Placebo	Male	70	Yes	Day 23	Yes	No	No	No
**2**	Placebo	Female	49	No	Day 7	No	No	No	No
**3**	Colchicine	Female	70	No	Day 11	No	No	No	No
**4**	Colchicine	Female	55	Yes	Day 7[b]	Yes	No	No	No
**5**	Colchicine	Male	43	No	Day 26	Yes	No	No	No
**6**	Colchicine	Male	46	Yes	Day 3	Yes	No	No	No
**7**	Colchicine	Male	57	Yes	Day 15	No	No	No	No
**8**	Colchicine	Male	63	Yes	Day 17	Yes	No	No	No
**9**	Colchicine	Male	77	No	Day 5	No	No	No	No
**10**	Colchicine	Female	50	Yes	Day 6	Yes	No	No	No
**11**	Colchicine	Male	62	No	Day 25	No	No	No	No
**12**	Colchicine	Female	50	Yes	Day 21[c]	No	No	No	No
**13**	Colchicine	Male	54	Yes	Day 13[d]	Yes	No	No	No

Abbreviations: MV, mechanical ventilation; PE, pulmonary embolus.

[a]Day of onset from randomization.

[b]Study medication stopped on Day 3.

[c]Study medication stopped on Day 15.

[d]Study medication stopped on Day 5.
